# Predictors of a Minimal Clinically Important Difference Following Omalizumab Treatment in Adult Patients With Severe Allergic Asthma

**DOI:** 10.3389/fmed.2021.762318

**Published:** 2022-01-03

**Authors:** Wei-Chang Huang, Pin-Kuei Fu, Ming-Cheng Chan, Chun-Shih Chin, Wen-Nan Huang, Kuo-Lung Lai, Jiun-Long Wang, Wei-Ting Hung, Yi-Da Wu, Chia-Wei Hsieh, Ming-Feng Wu, Yi-Hsing Chen, Jeng-Yuan Hsu

**Affiliations:** ^1^Division of Chest Medicine, Department of Internal Medicine, Taichung Veterans General Hospital, Taichung, Taiwan; ^2^College of Medicine, National Chung Hsing University, Taichung, Taiwan; ^3^Ph.D. Program in Translational Medicine, National Chung Hsing University, Taichung, Taiwan; ^4^School of Medicine, Chung Shan Medical University, Taichung, Taiwan; ^5^Master Program for Health Administration, Department of Industrial Engineering and Enterprise Information, Tunghai University, Taichung, Taiwan; ^6^Department of Medical Technology, Jen-Teh Junior College of Medicine, Nursing and Management, Miaoli, Taiwan; ^7^Department of Critical Care Medicine, Taichung Veterans General Hospital, Taichung, Taiwan; ^8^College of Human Science and Social Innovation, Hungkuang University, Taichung, Taiwan; ^9^Department of Computer Science, Tunghai University, Taichung, Taiwan; ^10^Division of Critical Care and Respiratory Therapy, Department of Internal Medicine, Taichung Veterans General Hospital, Taichung, Taiwan; ^11^Division of Allergy, Immunology and Rheumatology, Department of Internal Medicine, Taichung Veterans General Hospital, Taichung, Taiwan; ^12^Agricultural Biotechnology Research Center, National Chung Hsing University, Taichung, Taiwan; ^13^Department of Life Sciences, National Chung Hsing University, Taichung, Taiwan; ^14^Department of Medical Laboratory Science and Biotechnology, Central Taiwan University of Science and Technology, Taichung, Taiwan; ^15^School of Medicine, National Yang Ming Chia Tung University, Taipei, Taiwan; ^16^Division of Clinical Research, Department of Medical Research, Taichung Veterans General Hospital, Taichung, Taiwan; ^17^School of Physical Therapy, Chung-Shan Medical University, Taichung, Taiwan

**Keywords:** anti-IgE, asthma, minimal clinically important difference (MCID), omalizumab, predictor

## Abstract

Several factors have been found to be predictors of a good response following omalizumab treatment in patients with severe allergic asthma (SAA). However, it remains unclear whether clinical characteristics can predict a minimal clinically important difference (MCID) following omalizumab treatment in this population. Therefore, the aim of this study was to investigate the features associated with an MCID following omalizumab treatment in adult patients with SAA. Of the 124 participants enrolled in this retrospective, cross-sectional study, 94, 103, 20 and 53 achieved the MCID following treatment with omalizumab and were considered to be responders of exacerbation reduction (no exacerbation during the 1-year follow-up period or ≧50% reduction in exacerbations from baseline), oral corticosteroid (OCS) sparing (no use of OCS to control asthma during the study period or a reduction of the monthly OCS maintenance dose to <50% of baseline), lung function (an increase of ≧230 ml in the forced expiratory volume in 1 s from baseline) and asthma control (an increase of ≧3 points in the asthma control test score from baseline), respectively. Normal weight [<25 vs. ≧30 kg/m^2^, odds ratio (OR) = 3.86, *p* = 0.024] was predictive of a responder of reduction in exacerbations following omalizumab treatment while subjects with a blood eosinophil level of <300 cells/μL (<300 vs. ≧300 cells/μL, OR = 5.81, *p* = 0.001) were more likely to exhibit an MCID in OCS sparing. No factor was found to be a predictor of lung function or asthma control. When choosing treatment for adult patients with SAA, our findings may help to select those who may benefit the most from omalizumab treatment.

## Introduction

Asthma is a heterogeneous respiratory disease that involves airflow limitation due to chronic airway inflammation. It has been reported to affect 1–18% of the general population and is categorized into five Global Initiative for Asthma (GINA) steps (Step 1–5) based on the strength of treatment needed to control symptoms and exacerbations. Severe asthma, defined as asthma which cannot be controlled despite the use of GINA Step 4 or 5 pharmacological therapies, good inhaler technique and adherence, and optimal management of contributory factors, is estimated to occur in 3.6% of asthmatic patients (1, 2).

Omalizumab is an anti-immunoglobulin E (IgE) monoclonal antibody that reduces circulatory free IgE, and it is approved for the treatment of moderate to severe IgE-mediated asthma. The GINA recommendations suggest the use of omalizumab as add-on therapy for severe asthma owing to its noticeable impact on medical resource utilization, quality of life and beneficial treatment outcomes, including improved exacerbation rate, reduced oral corticosteroid (OCS) maintenance dose, better asthma control and improvements in lung function in both adult and pediatric patients with severe allergic asthma (SAA) (1, 3–10).

The statistical significance is the most widely used evidence to guide treatment decision making in both clinical trials and daily practice while this does not necessarily imply the clinical relevance. To overcome this gap, it is crucial to determine the minimal clinically important difference (MCID), first described in 1989 by Jaeschke et al. and defined as the smallest improvement in a treatment outcome considered worthwhile by an individual patient, for healthcare providers. Several MCID cut-off values have been proposed and validated in the population of asthma, with most of the cut-offs are associated with patient-reported outcomes, lung function and exercise tolerance (11–14). Nevertheless, the MCID has rarely used as a tool for assessing the treatment response of biologics for patients with severe asthma.

The GINA guidelines state that a blood eosinophil count ≧260 cells/μL, a fractional exhaled nitric oxide (FeNO) level ≧20 parts per billion, the presence of allergen-driven symptoms, and childhood-onset asthma are statistically significant predictors for a good therapeutic response to omalizumab in reducing exacerbations for patients with SAA (1). However, little is known about whether clinical characteristics are associated with the MCIDs, particularly those regarding the goals of asthma management proposed by the GINA strategy (1), following treatment with omalizumab in this population.

We hypothesized that the baseline clinical features could predict a worthwhile response to omalizumab as an add-on therapy for patients with SAA. Therefore, we investigated the pre-omalizumab treatment clinical characteristics associated with an MCID in reducing exacerbations, OCS sparing, and improvements in lung function and asthma control, the most representative and clinically vital treatment goals recommended by the GINA strategy (1), following treatment with omalizumab in adult patients with SAA.

## Materials and Methods

### Study Design, Setting, and Population

This retrospective cross-sectional study was approved by the Institutional Review Board and Ethics Committee of Taichung Veterans General Hospital (TCVGH) (Approval No. CE19015B) and implemented in accordance with the Declaration of Helsinki. The need for informed consent from participants was waived because of the retrospective nature of this study and data extraction based on an electronic medical chart review. The study was conducted at TCVGH, a tertiary referral center in central Taiwan, be-tween January 2010 and January 2019, and enrolled patients diagnosed of SAA who applied for reimbursements for omalizumab from the Taiwan National Health Insurance (NHI) according to the judgment of very experienced pulmonologists and immunologists in charge of asthma management. Patients whose applications were not ap-proved were excluded from this study (8).

### Data Collection

As detailed elsewhere (8), the investigators collected clinical data, including base-line demographics, clinical features and laboratory findings, medications related to asthma management, and co-morbidities for each participant. Moreover, the treatment outcomes of interest, including exacerbation history, usage of OCSs, spirometric data and asthma control test (ACT) scores were also recorded at baseline and the end of 1-year follow-up (8).

### Definition of MCID (Responder) According to Treatment Outcome of Interest

Responders with regards to a reduction in exacerbations were defined as those who had no exacerbations during the study period or who had a ≧50% reduction in the number of exacerbations in the 1-year follow-up period compared to the year prior to enrollment. An exacerbation was defined as a worsening of respiratory symptoms and lung function that required OCS treatment for ≧3 days at an outpatient service, emergency visit or hospitalization (15, 16). The patients who did not meet these criteria were defined as non-responders.

Responders with regards to OCS sparing were defined as those who did not use OCS to control asthma during the study period, or whose monthly OCS maintenance dose at the end of study was <50% compared to that at enrollment. Maintenance pharmacological therapy for SAA was defined as >7 days of OCS prescriptions in the outpatient department. The patients who did not meet these criteria were defined as non-responders.

The patients with an MCID according to lung function and ACT following omalizumab treatment were defined as those with an increase of ≧230 ml and ≧3 points in the forced expiratory volume in 1 s (FEV1) and ACT score, respectively, between the end and start of the 1-year follow-up period (13, 14). The patients who did not meet these criteria were defined as non-responders.

### Statistical Analysis

Categorical variables were presented as frequency and percentage and compared using the chi-squared test between the responders and non-responders. Continuous variables were presented using mean and standard deviation, and median and inter-quartile range, and were compared using a paired sample *t*-test or Wilcoxon signed-rank-test according to the normality assumption between the study groups. Logistic regression models were used to analyze independent factors if they were significant in the univariate analysis for patients with binary results of treatment outcomes of interest. A significant difference was defined as a two-sided *p*-value <0.05. The data of all patients were de-identified before analysis.

## Results

**Figure 1:**
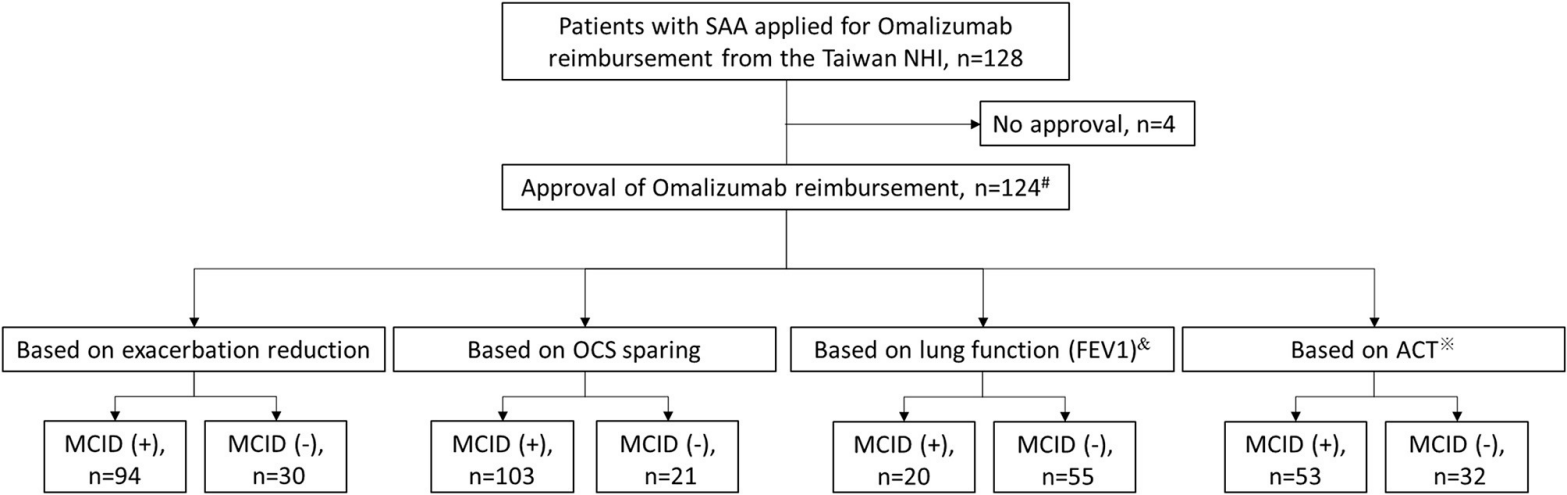
Patient enrolment flow chart. ^#^14 patients received omalizumab for only 4 months because of administrative issues from the Taiwan NHI, while the rest had at least 10 months of omalizumab treatment during the 1-year follow-up period. ^&^Only 75 patients had binary results of lung function measurement for analysis. ^※^Only 85 patients had binary results of ACT for analysis. ACT, asthma control test; FEV1, forced expiratory volume in 1 s; MCID, minimal clinically important difference; NHI, National Health Insurance; OCS, oral corticosteroid; SAA, severe allergic asthma.

Figure 1 shows the patient enrolment flow chart. Of the 128 patients with SAA who applied for reimbursements for omalizumab during the study period, 124 received approval and were included in the final analysis. Among the 124 enrollees, 110 patients received omalizumab treatment for at least 10 months during the 1-year follow-up period while 14 subjects received only 4 months of omalizumab because of the administrative issue from the Taiwan NHI (8). Only 75 and 85 patients had binary results of lung function measurements and ACT scores, respectively, at enrollment and the end of the 1-year follow-up period for further investigation. Of the 124 patients, 94 (75.8%), 103 (83.1%), 20 (26.7%), and 53 (62.4%) were identified as responders according to a reduction in exacerbations, OCS sparing, and improvements in lung function and asthma control, respectively.

**Figure 2:**
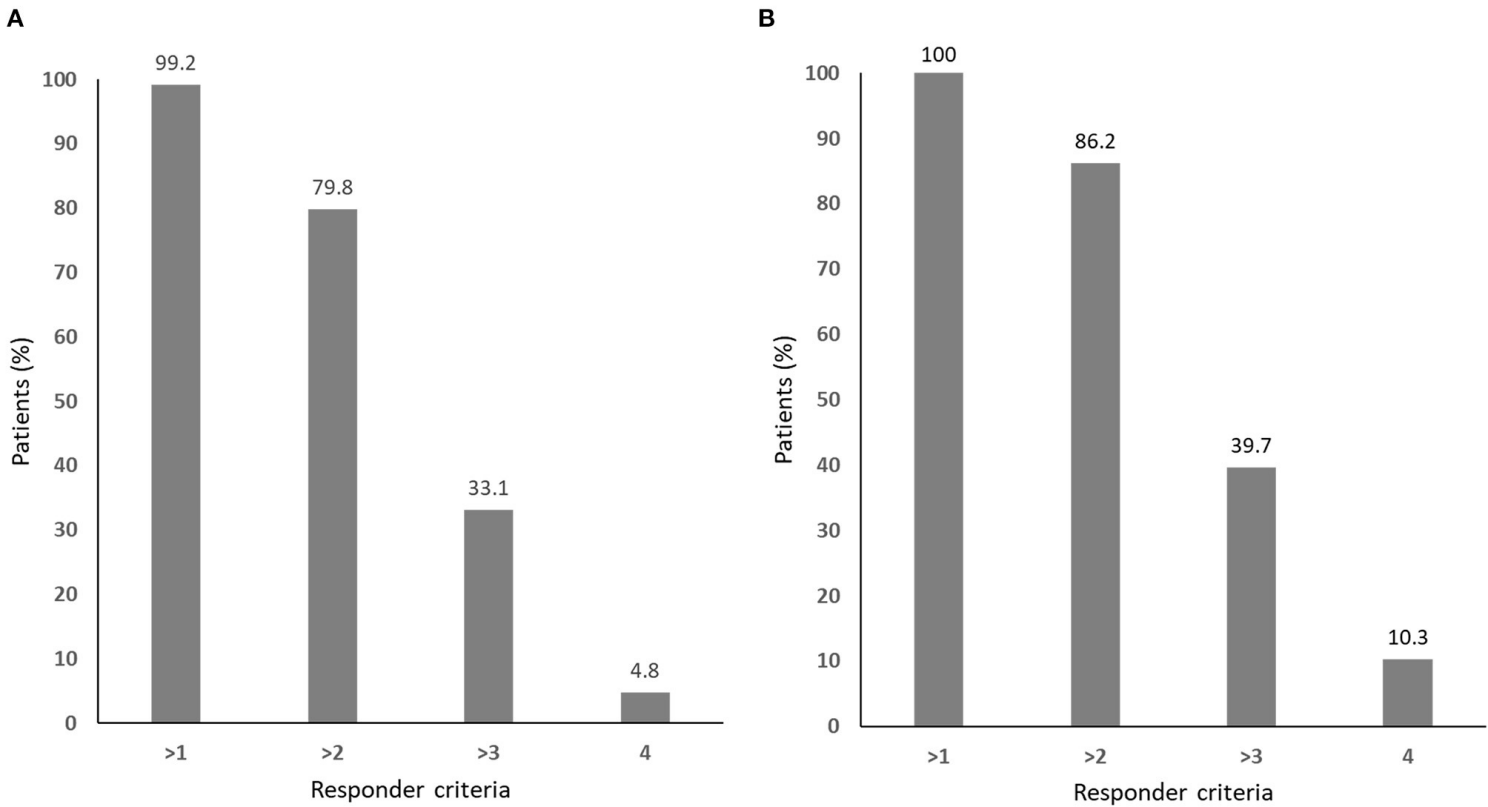
The responder distribution for **(A)** all patients (*n* = 124) and **(B)** those without missing values in any of the four responder criteria (*n* = 58).

Nearly all of the patients met one or more of the responder criteria, while only 4.8% of the patients with complete data and 10.3% of the those with missing values met all four responder criteria, respectively (Figure 2).

The mean age of the 124 patients was 60.8 ± 15.7 years (Table 1). More than a quarter of the patients had substantial smoking exposure, defined as ≧10 pack-years of cigarette smoking in their lifetime (35/124, 28.2%), while less than half were normal weight, de-fined as a body mass index <25 kg/m^2^ (53/124, 42.7%). Furthermore, of the 124 patients, 46 (37.1%) had blood eosinophilia of ≧300 cells/μL (Table 1).

**Table 1:** Baseline information of the enrolled participants and the responder analysis based on exacerbation reduction and oral corticosteroid sparing.

	**Exacerbation reduction**			**OCS sparing**			
	**Responder**	**Non-responder**	***p-*value**	**Responder**	**Non-responder**	***p-*value**	**Total**
	**(*n* = 94)**	**(*n* = 30)**		**(*n* = 103)**	**(*n* = 21)**		**(*n* = 124)**
**Age (years)**			0.303			0.886	
Mean ± SD	61.4 ± 15.9	58.9 ± 15.3		60.7 ± 16.0	61.6 ± 14.7		60.8 ± 15.7
Median (Q1, Q3)	64.0 (50.8, 74.0)	57.0 (48.5, 68.3)		62.0 (50.0, 71.0)	61.0 (50.5, 73.5)		62.0 (50.0, 71.0)
Male gender	49 (52.1%)	18 (60.0%)	0.587	56 (54.4%)	11 (52.4%)	1.000	67 (54.0%)
**BMI (kg/m** ^**2**^ **)** ^**#**^			0.032^*^			0.792	
Mean ± SD	25.8 ± 4.3	27.6 ± 4.5		26.2 ± 4.5	26.0 ± 4.2		26.2 ± 4.4
Median (Q1, Q3)	25.0 (22.5, 28.3)	26.4 (25.1, 30.1)		25.3 (23.3, 29.6)	25.6 (22.6, 27.5)		25.3 (23.1, 29.4)
<25	47 (50.0%)	6 (20.0%)		44 (42.7%)	9 (42.9%)		53 (42.7%)
≧25, <30	28 (29.8%)	15 (50.0%)		35 (34.0%)	8 (38.1%)		43 (34.7%)
≧30	19 (20.2%)	9 (30.0%)		24 (23.3%)	4 (19.0%)		28 (22.6%)
**Smoking (pack-year)**			0.046^*^			0.837	
Mean ± SD	7.3 ± 14.6	15.7 ± 28.0		9.9 ± 20.3	6.4 ± 10.1		9.3 ± 19.0
Median (Q1, Q3)	0.0 (0.0, 10.0)	0.0 (0.0, 22.5)		0.0 (0.0, 10.0)	0.0 (0.0, 20.0)		0.0 (0.0, 13.8)
≧10	24 (25.5%)	11 (36.7%)		29 (28.2%)	6 (28.6%)		35 (28.2%)
**Smoking history**			0.139			0.500	
Never smoker	66 (70.2%)	16 (53.3%)		68 (66.0%)	14 (66.7%)		82 (66.1%)
Ex-smoker	25 (26.6%)	11 (36.7%)		29 (28.2%)	7 (33.3%)		36 (29.0%)
Current smoker	3 (3.2%)	3 (10.0%)		6 (5.8%)	0 (0.0%)		6 (4.8%)
**Time for asthma history (years)**			0.407			0.934	
Mean ± SD	3.7 ± 3.6	4.0 ± 3.2		3.8 ± 3.6	3.7 ± 3.1		3.7 ± 3.5
Median (Q1, Q3)	2.6 (0.8, 5.4)	3.5 (0.9, 6.6)		2.9 (0.8, 5.7)	2.0 (0.9, 6.1)		2.8 (0.9, 5.8)
Total IgE (kU/L)			0.764			0.813	
Mean ± SD	750.3 ± 723.8	722.3 ± 757.4		725.2 ± 693.8	833.4 ± 896.4		743.5 ± 729.0
Median (Q1, Q3)	530.0 (259.0, 985.5)	464.5 (287.0, 856.8)		510.0 (269.0, 954.0)	472.0 (317.0, 1050.0)		507.5 (274.8, 968.3)
WBC (10^9^/L)			0.171			0.757	
Mean ± SD	8.1 ± 2.5	9.5 ± 3.9		8.5 ± 3.0	8.2 ± 2.7		8.5 ± 2.9
Median (Q1, Q3)	7.7 (6.4, 9.0)	7.9 (6.5, 12.6)		7.7 (6.5, 9.9)	8.0 (6.3, 9.0)		7.9 (6.5, 9.8)
**Blood absolute eosinophil count (cells/μL)**			0.117			0.003^*^	
Mean ± SD	422.4 ± 893.2	338.3 ± 539.8		362.5 ± 854.7	596.0 ± 606.1		402.1 ± 820.5
Median (Q1, Q3)	236.0 (129.3, 418.7)	135.8 (73.4, 443.3)		205.2 (109.3, 364.0)	518.4 (206.8, 793.6)		223.2 (111.3, 422.2)
≧300	36 (38.3%)	10 (33.3%)		31 (30.1%)	15 (71.4%)		46 (37.1%)
**Number of allergens tested**			1.000			0.245	
Mean ± SD	1.9 ± 1.6	1.9 ± 1.4		2.0 ± 1.5	1.6 ± 1.6		1.9 ± 1.5
Median (Q1, Q3)	2.0 (1.0, 3.0)	2.0 (1.0, 3.0)		2.0 (1.0, 3.0)	1.0 (0.0, 3.0)		2.0 (1.0, 3.0)
**Initial Omalizumab dose (mg/month)**			0.488			0.362	
Mean ± SD	447.7 ± 227.3	478.5 ± 232.4		465.8 ± 239.1	406.3 ± 161.4		455.4 ± 228.0
Median (Q1, Q3)	450.0 (300.0, 600.0)	450.0 (300.0, 600.0)		450.0 (300.0, 600.0)	450.0 (300.0, 525.0)		450.0 (300.0, 600.0)
**Inhaled medication**			1.000			0.475	
Medium-dose ICS/LABA ± Tiotropium	40 (42.6%)	13 (43.3%)		46 (44.7%)	7 (33.3%)		53 (42.7%)
High-dose ICS/LABA ± Tiotropium	54 (57.4%)	17 (56.7%)		57 (55.3%)	14 (66.7%)		71 (57.3%)
**Oral medication**			0.296			0.235	
None	7 (9.4%)	4 (13.3%)		10 (9.7%)	1 (4.8%)		11 (8.9%)
Montelukast alone	53 (56.4%)	20 (66.7%)		59 (57.3%)	14 (66.7%)		73 (58.9%)
Methylxanthines alone	12 (12.8%)	1 (3.3%)		9 (8.7%)	4 (19.0%)		13 (10.5%)
Montelukast + Methylxanthines	22 (23.4%)	5 (16.7%)		25 (24.3%)	2 (9.5%)		27 (21.8%)
**OCS maintenance dose (mg/month)**			0.351			0.173	
Mean ± SD	94.8 ± 191.8	98.0 ± 173.0		94.0 ± 194.6	103.3 ± 146.2		95.6 ± 186.8
Median (Q1, Q3)	0.0 (0.0, 140.0)	0.0 (0.0, 140.0)		0.0 (0.0, 140.0)	0.0 (0.0, 210.0)		0.0 (0.0, 140.0)
Early cessation of Xolair treatment	12 (12.8%)	2 (6.7%)	0.515	12 (11.7%)	2 (9.5%)	1.000	14 (11.3%)
**Co-morbidity**							
Depression	19 (20.2%)	4 (13.3%)	0.566	16 (15.5%)	7 (33.3%)	0.068	23 (18.5%)
Insomnia	21 (22.3%)	5 (16.7%)	0.684	22 (21.4%)	4 (19.0%)	1.000	26 (21.0%)
Osteoporosis	10 (10.6%)	2 (6.7%)	0.729	9 (8.7%)	3 (14.3%)	0.426	12 (9.7%)
Cerebrovascular disease	9 (9.6%)	5 (16.7%)	0.324	11 (10.7%)	3 (14.3%)	0.705	14 (11.3%)
GERD	26 (27.7%)	11 (36.7%)	0.478	30 (29.1%)	7 (33.3%)	0.903	37 (29.8%)
COPD	26 (27.7%)	10 (33.3%)	0.715	30 (29.1%)	6 (28.6%)	1.000	36 (29.0%)
DM	18 (19.1%)	5 (16.7%)	0.972	18 (17.5%)	5 (23.8%)	0.540	23 (18.5%)
Food or drug allergy	7 (7.4%)	3 (10.0%)	0.703	9 (8.7%)	1 (4.8%)	1.000	10 (8.1%)
Atopic disease^※^	82 (87.2%)	28 (93.3%)	0.515	91 (88.3%)	19 (90.5%)	1.000	110 (88.7%)
AERD	1 (1.1%)	0 (0.0%)	1.000	1 (1.0%)	0 (0.0%)	1.000	1 (0.8%)
OSAS	5 (5.3%)	0 (0.0%)	0.335	4 (3.9%)	1 (4.8%)	1.000	5 (4.0%)

* *p < 0.05.*

# *Categorized based on the World Health Organization recommendations.*

※ *Atopic disease included allergic dermatitis, allergic rhinitis, allergic conjunctivitis and food allergies.*

*AERD, aspirin-exacerbated respiratory disease; BMI, body mass index; COPD, chronic obstructive pulmonary disease; DM, diabetes mellitus; GERD, gastro-esophageal reflux disease; ICS, inhaled corticosteroid; IgE, immunoglobulin E; LABA, long-acting beta-agonist; OCS, oral corticosteroid; OSAS, obstructive sleep apnea syndrome; Q, quartile; SD, standard deviation; WBC, white blood count*.

**Figure 3:**
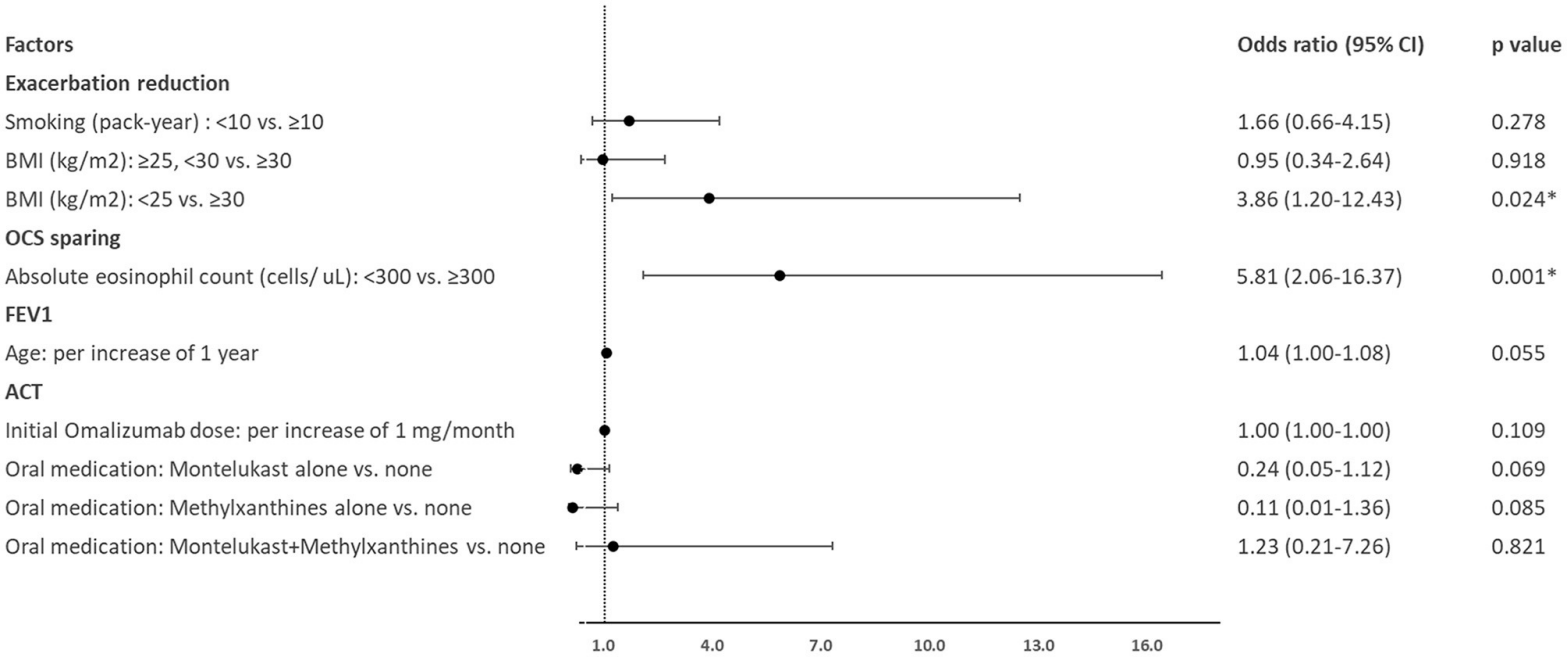
The factors associated with a minimal clinically important difference according to the treatment outcome of interest. ACT, asthma control test; BMI, body mass index; CI, confidence interval; FEV1, forced expiratory volume in 1 s; OCS, oral corticosteroid. *p < 0.05.

Compared to the non-responders regarding reduction in exacerbations, the responders had a significantly higher and lower proportion of normal weight and substantial smoking exposure, respectively (Table 1), while the former was independently associated with an MCID in a reduction in exacerbations (Figure 3).

Responders with OCS sparing had a lower blood eosinophil level expressed by absolute count (cells/μL) compared to the non-responders (Table 1). The logistic regression analysis showed that <300 cells/μL of circulating eosinophils was a significant predictor of an MCID in the sparing of OCS to control asthma (Figure 3).

The patients who exhibited an MCID in FEV1 improvement following omalizumab treatment were younger than those who did not (Table 2), although a younger age was not independently predictive of this treatment outcome (Figure 3).

**Table 2:** The factors associated with the responder of lung function improvement.

	**FEV1**			
	**Responder (*n* = 20)**	**Non-responder (*n* = 55)**	***p*-value**	**Total (*n* = 75)**
**Age (years)**			0.047^*^	
Mean ± SD	56.3 ± 17.5	63.8 ± 13.2		61.8 ± 14.7
Median (Q1, Q3)	54.5 (42.5, 71.5)	65.0 (55.0, 70.0)		63.0 (51.0, 70.0)
Male gender	12 (60.0%)	27 (49.1%)	0.565	39 (52.0%)
**BMI (kg/m** ^**2**^ **)**			0.848	
Mean ± SD	27.2 ± 5.3	26.6 ± 4.4		26.8 ± 4.6
Median (Q1, Q3)	26.0 (24.0, 30.5)	26.3 (23.4, 29.9)		26.3 (23.5, 30.2)
**Smoking (pack-year)**			0.875	
Mean ± SD	9.9 ± 13.8	10.4 ± 18.4		10.3 ± 17.2
Median (Q1, Q3)	0.0 (0.0, 20.0)	0.0 (0.0, 20.0)		0.0 (0.0, 20.0)
**Smoking history**			0.545	
Never smoker	12 (60.0%)	34 (61.8%)		46 (61.3%)
Ex-smoker	6 (30.0%)	19 (34.5%)		25 (33.3%)
Current smoker	2 (10.0%)	2 (3.6%)		4 (5.3%)
**Time for asthma history (years)**			0.679	
Mean ± SD	3.2 ± 2.8	4.2 ± 4.2		3.9 ± 3.9
Median (Q1, Q3)	2.3 (0.8, 5.2)	2.8 (0.7, 6.6)		2.8 (0.8, 6.5)
**Total IgE (kU/L)**			0.774	
Mean ± SD	614.2 ± 443.3	670.0 ± 530.0		655.1 ± 506.0
Median (Q1, Q3)	425.0 (311.0, 836.8)	532.0 (219.0, 875.0)		510.0 (289.0, 860.0)
**WBC (10** ^**9**^ **/L)**			0.679	
Mean ± SD	9.0 ± 2.9	8.8 ± 3.4		8.9 ± 3.2
Median (Q1, Q3)	8.2 (6.6, 11.9)	7.9 (6.4, 10.6)		7.9 (6.5, 10.8)
**Blood absolute eosinophil count (cells/μL)**			0.375	
Mean ± SD	496.6 ± 671.3	319.2 ± 427.6		366.5 ± 505.3
Median (Q1, Q3)	265.7 (111.8, 576.1)	207.9 (111.2, 352.8)		220.0 (111.7, 384.0)
**Number of allergens tested**			0.686	
Mean ± SD	2.1 ± 1.7	1.9 ± 1.4		1.9 ± 1.5
Median (Q1, Q3)	2.0 (1.0, 3.0)	2.0 (1.0, 3.0)		2.0 (1.0, 3.0)
**Initial omalizumab dose (mg/month)**			0.725	
Mean ± SD	443.4 ± 156.8	435.6 ± 226.2		437.7 ± 208.9
Median (Q1, Q3)	450.0 (300.0, 600.0)	412.5 (300.0, 600.0)		450.0 (300.0, 600.0)
**Inhaled medication**			0.522	
Medium-dose ICS/LABA ± Tiotropium	4 (20.0%)	17 (30.9%)		21 (28.0%)
High-dose ICS/LABA ± Tiotropium	16 (80.0%)	38 (69.1%)		54 (72.0%)
**Oral medication**			0.833	
None	2 (10.0%)	6 (10.9%)		8 (10.7%)
Montelukast alone	14 (70.0%)	33 (60.0%)		47 (62.7%)
Methylxanthines alone	2 (10.0%)	6 (10.9%)		8 (10.7%)
Montelukast + Methylxanthines	2 (10.0%)	10 (18.2%)		12 (16.0%)
Initial OCS maintenance dose (mg/month)			0.519	
Mean ± SD	87.5 ± 191.2	64.0 ± 129.3		70.3 ± 147.3
Median (Q1, Q3)	0.0 (0.0, 140.0)	0.0 (0.0, 140.0)		0.0 (0.0, 140.0)
Early cessation of Xolair treatment	2 (10.0%)	5 (9.1%)	1.000	7 (9.3%)
**Co-morbidity**				
Depression	1 (5.0%)	8 (14.5%)	0.430	9 (12.0%)
Insomnia	2 (10.0%)	12 (21.8%)	0.328	14 (18.7%)
Osteoporosis	2 (10.0%)	5 (9.1%)	1.000	7 (9.3%)
Cerebrovascular disease	1 (5.0%)	5 (9.1%)	1.000	6 (8.0%)
GERD	7 (35.0%)	17 (30.9%)	0.955	24 (32.0%)
COPD	4 (20.0%)	19 (34.5%)	0.355	23 (30.7%)
DM	2 (10.0%)	9 (16.4%)	0.717	11 (14.7%)
Food or drug allergy	3 (15.0%)	2 (3.6%)	0.114	5 (6.7%)
Atopic disease^※^	19 (95.0%)	49 (89.1%)	0.667	68 (90.7%)
AERD	1 (5.0%)	0 (0.0%)	0.267	1 (1.3%)
OSAS	0 (0.0%)	3 (5.5%)	0.560	3 (4.0%)

* *p < 0.05.*

※ *Atopic disease included allergic dermatitis, allergic rhinitis, allergic conjunctivitis and food allergies.*

*FEV1, forced expiratory volume in 1 s; also see Table 1*.

The responders with an improvement in ACT following omalizumab treatment were associated with a higher initial dose of omalizumab and more use of either montelukast alone or methylxanthines alone to control asthma (Table 3). None of these characteristics could independently predict an MCID in an improvement in ACT score (Figure 3).

**Table 3:** The responder analysis for the asthma control improvement.

	**ACT**			
	**Responder (*n* = 53)**	**Non-responder (*n* = 32)**	***p-*value**	**Total (*n* = 85)**
**Age (years)**			0.993	
Mean ± SD	63.0 ± 14.6	62.6 ± 15.4		62.9 ± 14.8
Median (Q1, Q3)	62.0 (52.5, 74.5)	64.0 (51.3, 76.5)		63.0 (51.5, 75.5)
Male gender	28 (52.8%)	21 (65.6%)	0.352	49 (57.6%)
**BMI (kg/m** ^**2**^ **)**			0.910	
Mean ± SD	26.9 ± 4.8	26.9 ± 4.1		26.9 ± 4.5
Median (Q1, Q3)	26.5 (22.8, 30.3)	26.0 (23.8, 29.9)		26.3 (23.4, 30.0)
**Smoking (pack-year)**			0.209	
Mean ± SD	11.8 ± 24.5	11.7 ± 16.7		11.8 ± 21.8
Median (Q1, Q3)	0.0 (0.0, 20.0)	0.5 (0.0, 20.0)		0.0 (0.0, 20.0)
**Smoking history**			0.220	
Never smoker	36 (67.9%)	16 (50.0%)		52 (61.2%)
Ex-smoker	15 (28.3%)	13 (40.6%)		28 (32.9%)
Current smoker	2 (3.8%)	3 (9.4%)		5 (5.9%)
**Time for asthma history (years)**			0.083	
Mean ± SD	3.4 ± 3.6	4.3 ± 3.1		3.7 ± 3.4
Median (Q1, Q3)	1.9 (0.6, 5.0)	4.1 (1.5, 6.6)		2.9 (0.8, 6.1)
**Total IgE (kU/L)**			0.116	
Mean ± SD	878.8 ± 886.4	605.2 ± 638.7		775.8 ± 809.2
Median (Q1, Q3)	538.0 (326.0, 1026.0)	431.5 (205.5, 847.8)		505.0 (285.0, 903.0)
**WBC (10** ^**9**^ **/L)**			0.098	
Mean ± SD	8.2 ± 3.1	9.2 ± 3.1		8.6 ± 3.1
Median (Q1, Q3)	7.5 (5.9, 9.9)	8.3 (7.1, 11.3)		7.9 (6.4, 10.5)
**Blood absolute eosinophil count (cells/μL)**			0.608	
Mean ± SD	304.0 ± 285.5	391.5 ± 538.8		336.9 ± 399.2
Median (Q1, Q3)	226.1 (114.7, 378.8)	248.0 (111.4, 412.7)		241.7 (111.6, 394.7)
**Number of allergens tested**			0.934	
Mean ± SD	2.0 ± 1.6	2.1 ± 1.7		2.1 ± 1.6
Median (Q1, Q3)	2.0 (1.0, 3.0)	2.0 (1.0, 3.0)		2.0 (1.0, 3.0)
**Initial Omalizumab dose (mg/month**)			0.041^*^	
Mean ± SD	488.5 ± 193.3	408.6 ± 240.2		459.2 ± 213.7
Median (Q1, Q3)	550.0 (300.0, 600.0)	300.0 (300.0, 600.0)		450.0 (300.0, 600.0)
**Inhaled medication**			0.331	
Medium-dose ICS/LABA ± Tiotropium	20 (37.7%)	8 (25.0%)		28 (32.9%)
High-dose ICS/LABA ± Tiotropium	33 (62.3%)	24 (75.0%)		57 (67.1%)
**Oral medication**			0.020^*^	
None	4 (7.5%)	5 (15.6%)		9 (10.6%)
Montelukast alone	37 (69.8%)	15 (46.9%)		52 (61.2%)
Methylxanthines alone	6 (11.3%)	1 (3.1%)		7 (8.2%)
Montelukast + Methylxanthines	6 (11.3%)	11 (34.4%)		17 (20.0%)
**Initial OCS maintenance dose (mg/month)**			0.288	
Mean ± SD	50.2 ± 89.1	127.5 ± 233.2		79.3 ± 162.5
Median (Q1, Q3)	0.0 (0.0, 122.5)	0.0 (0.0, 140.0)		0.0 (0.0, 140.0)
Early cessation of Xolair treatment	6 (11.3%)	3 (9.4%)	1.000	9 (10.6%)
**Co-morbidity**				
Depression	9 (17.0%)	5 (15.6%)	1.000	14 (16.5%)
Insomnia	13 (24.5%)	5 (15.6%)	0.484	18 (21.2%)
Osteoporosis	4 (7.5%)	4 (12.5%)	0.468	8 (9.4%)
Cerebrovascular disease	6 (11.3%)	3 (9.4%)	1.000	9 (10.6%)
GERD	16 (30.2%)	11 (34.4%)	0.872	27 (31.8%)
COPD	14 (26.4%)	15 (46.9%)	0.091	29 (34.1%)
DM	12 (22.6%)	3 (9.4%)	0.207	15 (17.6%)
Food or drug allergy	5 (9.4%)	2 (6.3%)	0.706	7 (8.2%)
Atopic disease^※^	50 (94.3%)	26 (81.3%)	0.075	76 (89.4%)
AERD	0 (0.0%)	0 (0.0%)	NA	0 (0.0%)
OSAS	4 (7.5%)	0 (0.0%)	0.292	4 (4.7%)

* *p < 0.05.*

※ *Atopic disease included allergic dermatitis, allergic rhinitis, allergic conjunctivitis and food allergies.*

*ACT, asthma control test; also see Table 1*.

## Discussion

In this study of 124 adult patients with SAA, 75.8, 83.1, 26.7, and 62.4% were considered to be responders following omalizumab treatment according to a reduction in exacerbations, OCS sparing, and improvements in lung function and asthma control, respectively. The responders with a reduction in exacerbations were characterized by normal weight and less smoking exposure; OCS sparing by a lower blood eosinophil level; lung function improvement by a younger age; and asthma control improvement by a higher initial dose of omalizumab and more use of either montelukast alone or methylxanthines alone to control asthma. In addition, normal weight was a significant predictor of an MCID in a reduction in exacerbations following omalizumab treatment; a circulatory eosinophil level of <300 cells/μL in OCS sparing; and none in improvements in lung function or asthma control.

Our results showed that normal weight and less smoking exposure were associated with an MCID in a reduction in the annual number of exacerbations, and the former was an independent predictor. In contrast to our study, Casale et al. enrolled a relatively young population (a mean age of 47.3 years) with allergic asthma who were candidates for omalizumab treatment, and found that those with an increased number of exacerbations in the year prior to the study as well as female patients were more likely to be responders with a reduction in exacerbations defined using a similar criteria to our study (5, 17). The inconsistencies in the features between our study and Casale et al.'s may mainly be due to the disparity in age of the study populations (a mean age of 60.8 vs. 47.3 years) (5). In addition, previous studies have reported that obesity, co-morbidities and the presence of nasal polyps were risk factors for exacerbations, while more clinically severe asthma (as evaluated by emergency visits and hospitalizations for asthma in the previous year, FEV1 % predicted <65 vs. ≥65, inhaled beclomethasone dipropionate dose <600 vs. ≥600 μg per day, and long-acting beta-agonist use vs. non-use) and high Th2-driven inflammatory biomarkers (including FeNO, peripheral blood eosinophils, and serum periostin and total IgE) were associated with a greater reduction in exacerbations following treatment with omalizumab (4, 6, 18, 19).

We also found that a peripheral blood eosinophil level of <300 cells/μL could predict responders with OCS sparing. Hanania et al. reported that a high level of FeNO could predict less albuterol use (4). In contrast, Sposato et al. reported that obesity was independently associated with the excessive use of short-acting beta-agonists and increased dose of medications to control asthma, and that nasal polyps were associated with the use of a higher inhaler corticosteroid dose (18).

Further, our study showed that the responders with lung function improvement were younger compared to the non-responders, and that the patients with a higher initial omalizumab dose and more use of oral medications to control asthma were associated with an MCID in asthma control. In contrast, Casale et al. reported that patients with uncontrolled asthma and receiving asthma medications in addition to inhaled corticosteroids (ICS)/long-acting beta-agonists were more likely to be associated with an MCID in lung function, defined as a rise in FEV1 ≧120 ml from baseline, while those with higher baseline circulatory eosinophil levels were more likely to be responders with asthma control according to a definition similar to ours (5). Again, the discrepancy in the characteristics between these two studies may be explained by the difference in age of the study population (a mean age of 60.8 vs. 47.3 years) and in the definition of a response in lung function (an FEV1 improvement of ≧230 vs. ≧120 ml) (5, 17). Conversely, Sposato et al. reported that patients had a reduced response following omalizumab treatment in both lung function and asthma control if they were older or obese, and had co-morbidities, particularly chronic heart disease (18). Taken together, these findings suggest that the SAA patients who had an MCID in the treatment outcomes of interest following omalizumab treatment had a predictable clinical behavior, although the discrepancies in age and in the definition of a responder led to differences in the predictors.

Obese patients with asthma have an increased risk of severe disease, which may arise from many factors, such as changes in airway anatomy, adipokines, glucose-insulin metabolism, oxidative stress, inflammation, and genetic and epigenetic variants (20). Furthermore, similar to our finding that the SAA patients with normal weight were more likely to be responders with a reduction in exacerbations, previous reports have shown that obesity may reduce ICS response and negatively influence the beneficial effect of omalizumab in terms of asthma control in patients with asthma (1, 21). These findings show that obesity has a great impact on the severity and prognosis of asthma, and that obesity is a particular phenotype of asthma.

The predictive value of total IgE and blood eosinophil levels with regards to the therapeutic benefit of omalizumab in patients with allergic asthma has yet to be shown in previous studies, although we found that a blood eosinophil level of <300 cells/μL was predictive of an MCID in OCS sparing in the SAA patients (7, 19, 22). This disparity could be explained by the use of different treatment outcomes for analysis between the present study and other studies (OCS sparing vs. reduction in exacerbations).

Similar to the response rates of 75.8 and 62.4% for a reduction in exacerbations and improvement in asthma control, respectively, Casale et al. reported response rates of 77.8 and 64.7% (5). However, we found a lower response rate of 26.7% for an improvement in lung function defined as an increase in FEV1 ≧230 ml between enrollment and the end of the study compared to 35.9% defined as a ≧120 ml improvement in FEV1 between the end and start of the study reported by Casale et al. (5, 13, 23). As expected, the definition of a responder affected the difference in response rate of treatment outcomes of interest in the asthmatic patients.

The main strength of the current study is that the diagnosis of SAA was made according to the GINA recommendations by clinicians who were actively involved in the management of asthma, while the initiation of omalizumab treatment in all patients was suggested by both the physician in charge and the Taiwan NHI committee for SAA (1), This ensured a valid study population of patients with SAA and reached a strong consensus on whether or not omalizumab should be an add-on therapy for SAA patients, although 14 of the 124 participants discontinued omalizumab treatment after 4 months because of administrative issues with the Taiwan NHI. This may compensate for the limitations of this study, which include the incomplete binary results of lung function tests and ACT data, older age of the participants, and the small number of cases. Due to these limitations, our results should be interpreted with caution, and they may not be generalizable to a younger population.

## Conclusions

It is extremely important to take clinical features into consideration when managing patients with asthma at different GINA steps, particularly at GINA step 5, because several inflammatory biomarkers used to predict a good therapeutic response to the biologic treatment, such as FeNO and periostin, are not always available in all hospitals or areas/countries. We identified several clinical characteristics associated with an MCID in terms of a reduction in exacerbations, OCS sparing, and improvements in lung function and asthma control in adult patients with SAA. This information could help when selecting patients who may benefit more from omalizumab treatment to manage asthma. Future well-designed studies including more subjects and more potential and easily-obtained inflammatory biomarkers are warranted to more accurately predict an MCID following omalizumab treatment.

## Data Availability Statement

The original contributions presented in the study are included in the article/supplementary material, further inquiries can be directed to the corresponding author.

## Ethics Statement

The studies involving human participants were reviewed and approved by the Institutional Review Board and Ethics Committee of Taichung Veterans General Hospital (Approval No. CE19015B). Written informed consent for participation was not required for this study in accordance with the national legislation and the institutional requirements.

## Author Contributions

W-CH: conceptualization, methodology, formal analysis, investigation, data curation, writing—original draft preparation, visualization, project administration, and agreement on the published version of the manuscript. P-KF, M-CC, C-SC, W-NH, K-LL, J-LW, W-TH, Y-DW, C-WH, and M-FW: methodology, formal analysis, investigation, data curation, writing—review and editing, visualization, and agreement on the published version of the manuscript. Y-HC and J-YH: conceptualization, methodology, validation, formal analysis, investigation, data curation, writing—review and editing, visualization, supervision, and agreement on the published version of the manuscript. All authors contributed to the article and approved the submitted version.

## Conflict of Interest

The authors declare that the research was conducted in the absence of any commercial or financial relationships that could be construed as a potential conflict of interest.

## Publisher's Note

All claims expressed in this article are solely those of the authors and do not necessarily represent those of their affiliated organizations, or those of the publisher, the editors and the reviewers. Any product that may be evaluated in this article, or claim that may be made by its manufacturer, is not guaranteed or endorsed by the publisher.
